# Interleukin-2 Receptor Antagonist Induction Therapy in Lung Transplantation—A Meta-Analysis of Reconstructed Time-to-Event Data

**DOI:** 10.3390/jcm15041438

**Published:** 2026-02-12

**Authors:** Felipe S. Passos, Erlon de Avila Carvalho, Rachid E. Oliveira, Ricardo E. Treml, Hristo Kirov, Torsten Doenst, Bernardo M. Pessoa, Tulio Caldonazo

**Affiliations:** 1 Department of Thoracic Surgery, Mater Dei Hospital, Salvador 40221-500, Brazil; felipesanpassos@hotmail.com; 2 Hospital das Clínicas, Federal University of Minas Gerais, Belo Horizonte 30130-100, Brazil; erlon.avila@gmail.com; 3 Department of Thoracic Surgery, Barretos Cancer Hospital, Barretos 14784-400, Brazil; rachidccrr@hotmail.com; 4 Department of Anesthesiology, Perioperative and Pain Medicine, Stanford School of Medicine, Stanford, CA 94305, USA; rictreml@stanford.edu; 5 Department of Cardiothoracic Surgery, Jena University Hospital, 07747 Jena, Germany; hristo.kirov@med.uni-jena.de (H.K.); doenst@med.uni-jena.de (T.D.); 6 Department of Thoracic Surgery, Hospital Santa Cruz, Rede D’Or, Curitiba 80420-090, Brazil; pessoa.ber@gmail.com

**Keywords:** interleukin-2 receptor antagonists, basiliximab, daclizumab, lung transplantation, induction therapy

## Abstract

**Objectives:** Lung transplantation is a life-saving option for patients with end-stage lung diseases, yet immunosuppression management remains challenging. Induction therapy with interleukin-2 receptor antagonists (IL2-AR), such as basiliximab and daclizumab, is designed to reduce acute rejection and improve graft survival. However, its efficacy compared with alternative agents or no induction therapy remains uncertain. This study aimed to evaluate the impact of IL2-AR induction on clinical outcomes in lung transplant recipients. **Methods:** A systematic review and meta-analysis were conducted following PRISMA guidelines. Studies comparing IL2-AR induction with antithymocyte globulin (ATG), alemtuzumab, or no induction therapy were included. The primary outcomes were overall survival and freedom from acute rejection. Secondary outcomes included freedom from bronchiolitis obliterans syndrome (BOS), hospital length of stay (LOS), and time until extubation. Kaplan–Meier curves were reconstructed for long-term outcomes. Random effects model was performed. **Results:** Twelve studies comprising 27,855 patients were included. IL2-AR induction was associated with improved overall survival compared to standard of care (HR 0.88; 95%CI 0.85–0.93; *p* < 0.01). However, sensitivity analyses, including two-stage meta-analysis and leave-one-out analysis, revealed a loss of statistical significance. No significant differences were found for freedom from acute rejection (*p* = 0.774) or secondary outcomes, including freedom from BOS (*p* = 0.455), hospital LOS (*p* = 0.423), and time until extubation (*p* = 0.186). **Conclusions:** IL2-AR therapy may be associated with improved survival after lung transplantation; however, evidence remains inconclusive due to heterogeneity and limitations in study design.

## 1. Introduction

Lung transplantation is the definitive therapeutic option for selected patients with end-stage lung diseases [1,2]. Over recent decades, advances in donor selection, organ preservation, and peri- and postoperative management have significantly improved outcomes [3,4]. Despite this progress, lung transplantation continues to have the poorest long-term survival among solid organ transplants, largely due to the inherent complexity of immunological interactions between donor and recipient. Primary graft dysfunction (PGD), characterized by local graft inflammation leading to graft failure, remains the leading cause of early morbidity and mortality. Additionally, systemic inflammatory response syndrome (SIRS) and subsequent multi-organ dysfunction further complicate the clinical course, making management particularly challenging [5,6].

In this context, induction therapy has emerged as a promising strategy, demonstrating potential to effectively modulate the immune response in lung transplant recipients [6,7]. By attenuating T-cell activation during the critical early post-transplant period, induction agents aim to reduce the risk of acute cellular rejection and improve long-term graft survival [6]. Among the available agents, interleukin-2 receptor antagonists (IL2-AR), such as daclizumab and basiliximab, have gained attention due to their targeted mechanism of action, which specifically blocks IL-2 mediated T-cell activation [8]. This approach theoretically reduces the risks associated with broad immunosuppression, such as disseminated infections [9].

Despite their widespread use, there remains no consensus regarding the optimal induction strategy in lung transplantation. Clinical practices vary substantially across centers, and the available evidence is largely derived from observational studies with heterogeneous designs, limited sample sizes, and inconsistent reporting of long-term outcomes. Consequently, the true impact of IL2-AR induction on survival and rejection-free outcomes remains unclear [5,10]. Therefore, this systematic review and meta-analysis aimed to evaluate the efficacy and clinical outcomes of IL2-AR induction therapy in lung transplant recipients, comparing its impact with alternative induction agents, such as antithymocyte globulin and alemtuzumab, as well as with no induction therapy.

## 2. Methods

This systematic review and meta-analysis followed the Preferred Reporting Items for Systematic Reviews and Meta-analyses (PRISMA) guidelines [11] and the Cochrane Handbook of Systematic Reviews of Interventions. The study protocol was registered in the International Prospective Register of Systematic Reviews (PROSPERO, CRD CRD42024621991). Patient consent was not required as this study synthesized and analyzed data from previously published research.

### 2.1. Search Strategy

A comprehensive literature search was conducted on MEDLINE, EMBASE and the Cochrane Library to identify contemporary studies comparing outcomes between IL2-AR and other agents as induction therapy or no induction therapy in patients following lung transplantation, published up to September 2025. Reference lists of eligible articles were also screened to identify additional relevant studies. The full search strategy is detailed in Supplementary Table S1.

### 2.2. Study Selection

Two independent reviewers (FP and RENNO) screened the records after duplication removal. Any disagreements were resolved through discussion and consensus, with final decisions made by a third author (TC). Titles and abstracts were assessed according to predefined inclusion and exclusion criteria.

### 2.3. Eligibility Criteria

Inclusion criteria for studies involved in this analysis were as follows: (I) randomized controlled trials (RCTs) or observational studies; (II) comparing IL2-AR induction therapy with the institution’s standard of care (SOC) induction protocols, such as ATG, Alemtuzumab, OKT3, or no induction therapy; (III) enrolling adult patients undergoing lung transplantation; and (IV) reporting at least one outcome of interest. Exclusion criteria included studies involving animal models, conference abstracts, case reports, non-comparative study designs and those involving the use of IL2-AR prior to transplantation. For studies with overlapping populations, the one with the largest sample size was selected to minimize redundancy.

### 2.4. Quality Assessment and Publication Bias

Study quality was assessed using the ROBINS-I tool for non-randomized studies [12], categorizing risk of bias as critical, serious, moderate, or low across seven domains. Although Mullen et al. [13]. is an RCT, its small sample size led to its assessment using ROBINS-I to maintain consistency in the risk-of-bias evaluation across the meta-analysis. Publication bias was evaluated for the primary outcome.

### 2.5. Data Extraction

Data extraction was independently performed by two reviewers (FP and EAC), with accuracy verified by a third author (TC). Extracted data included study characteristics (year of publication, study period, country, sample size, intervention and comparator groups, and reported outcomes) and patient demographics (age, sex, and type of transplant).

### 2.6. Outcomes

The primary outcome was overall survival. The secondary outcomes included freedom from acute rejection, freedom from bronchiolitis obliterans syndrome (BOS), time until extubation and hospital length of stay (LOS). In acute rejection analysis, were considered significant rejection patients presenting acute cellular rejection grade ≥ A2 in the ISHLT criteria [14].

### 2.7. Statistical Analysis

Mean Difference (MD) values with 95% confidence intervals (CI) were calculated for continuous outcomes. A time-to-event data strategy was utilized for overall survival, freedom from acute rejection and freedom from BOS. Heterogeneity was assessed using the Cochran Q test and the I^2^ statistic with *p* < 0.10 and I^2^ > 50% considered indicative of significant heterogeneity [15]. A *p*-value < 0.05 was used to define statistical significance between groups. Random-effects models using the restricted maximum likelihood (REML) method were applied to account for interstudy variability and to provide more generalizable effect estimates across all analyzed outcomes.

To assess the robustness of our findings for the primary endpoint, we conducted several sensitivity analyses, including a two-stage meta-analysis using study-specific HRs, a leave-one-out analysis, and Egger’s test to evaluate publication bias.

Data handling and conversions were performed in accordance with the Cochrane Handbook for Systematic Reviews of Interventions [16]. All statistical analyses were conducted using R software, version 4.4.0 (R Foundation for Statistical Computing, Vienna, Austria), and Stata IC version 17.0 (StataCorp LLC, College Station, TX, USA).

### 2.8. Meta-Analysis of Reconstructed Data—One-Stage Survival Meta-Analysis

Time-to-event data for long-term outcomes were reconstructed using the methodology described by Wei et al. [17], which allows the approximation of individual patient data from published Kaplan–Meier curves. Raster and vector images of the survival curves were first preprocessed to optimize resolution and contrast and the subsequently digitized using specialized software to extract survival probabilities at predefined time points across the entire follow-up period.

When available, Supplementary Information reported in the original studies, such as the number of patients at risk at specific time points and the total number of events, was incorporated into the reconstruction process to refine the estimation of censoring patterns and event times, thereby improving the calibration and internal consistency of the reconstructed datasets. The accuracy of the reconstructed failure-time distributions was assessed by visually and quantitatively comparing the reconstructed Kaplan–Meier curves with the published survival or mortality curves and reported summary statistics from the original studies.

The Kaplan–Meier method [18] was used to calculate the overall survival, freedom from acute rejection, and freedom from BOS. The Cox proportional hazards regression model was used to assess between-group differences. For these Cox models, the proportional hazards assumption was verified by plotting scaled Schoenfeld residuals, log–log survival plots, and predicted versus observed survival functions. We plotted survival curves using the Kaplan–Meier product limit method and calculated the Hazard Ratios (HRs) and 95% CIs of each group.

The proportional hazards assumption for all Cox models was formally assessed using multiple complementary approaches, including visual inspection of scaled Schoenfeld residuals, evaluation of log–log survival plots, and comparison of predicted versus observed survival functions over time. Kaplan–Meier survival curves were generated for graphical comparison between groups, and all time-to-event analyses were conducted using the reconstructed individual-level data derived from the published studies.

## 3. Results

### 3.1. Study Characteristics

**Figure 1:**
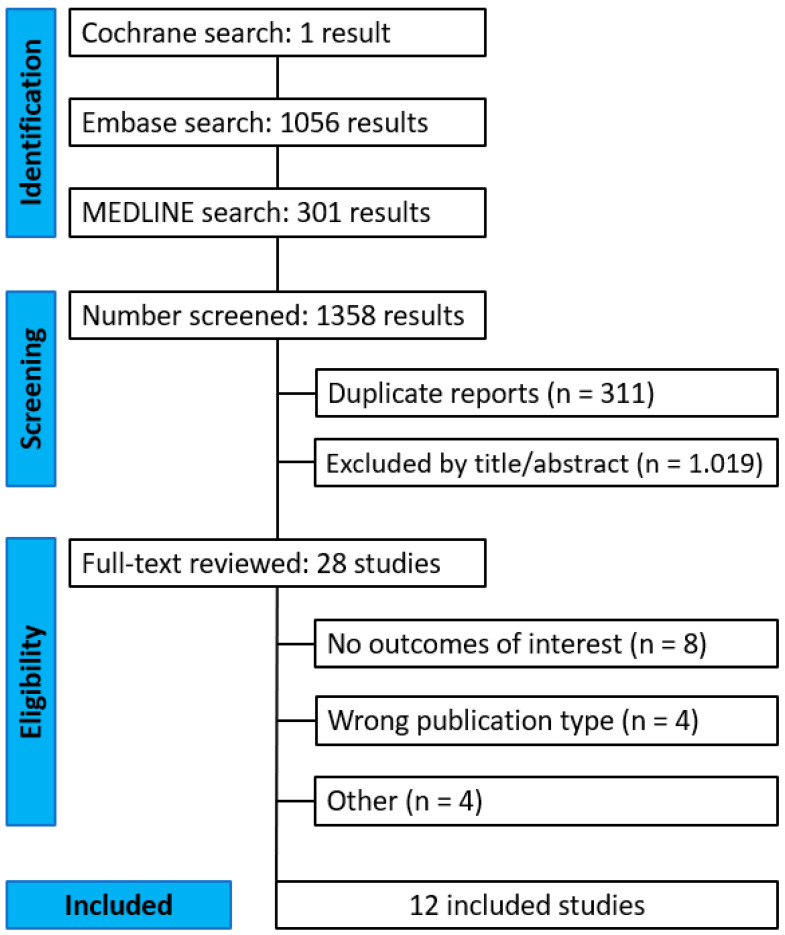
Preferred Reporting Items for Systematic Reviews and Meta-Analyses (PRISMA) flow diagram.

Figure 1 illustrates the PRISMA flow diagram summarizing the study selection process. Of the 1358 records identified through the systematic search, 12 studies met the eligibility criteria and were included in the final analysis [6,8,13,19,20,21,22,23,24,25,26,27]. Included studies were published between 2001 and 2022. All studies used data originated from Canada, Czech Republic, Denmark, Netherlands, Portugal and the United States of America. Table 1 summarizes the characteristics of the included studies.

### 3.2. Quality Assessment and Publication Bias

Risk of bias for overall survival was assessed using the ROBINS-I tool by two independent reviewers (Supplementary Figure S1). Overall, the included studies demonstrated a moderate risk of bias, with some domains showing lower risk. The primary sources of bias were related to confounding, outcome measurement, and selection of the reported results.

### 3.3. Patient Characteristics

Twelve studies (9 retrospective, 2 prospective, and 1 RCT) were included in this meta-analysis, encompassing 27,855 patients. Among these, 13,035 patients received IL2-AR, 4784 underwent an alternative induction therapy protocol and 10,036 did not receive induction therapy. The age ranged from 40 to 61 years, with the percentage of male patients varying from 37% to 95%. In the IL2-AR group, double lung transplantation was performed in 26.5% to 86.4% of patients, compared to 12.3% to 87.2% in the SOC group.

### 3.4. Primary Outcomes

**Figure 2:**
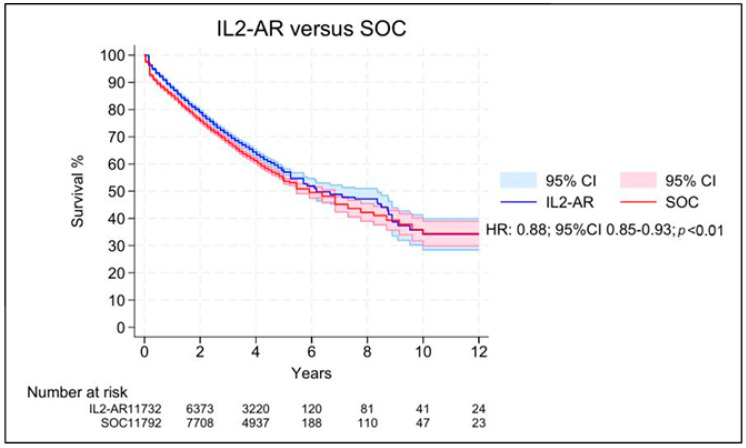
Overall survival for patients receiving IL2–AR versus SOC as induction therapy.

**Figure 3:**
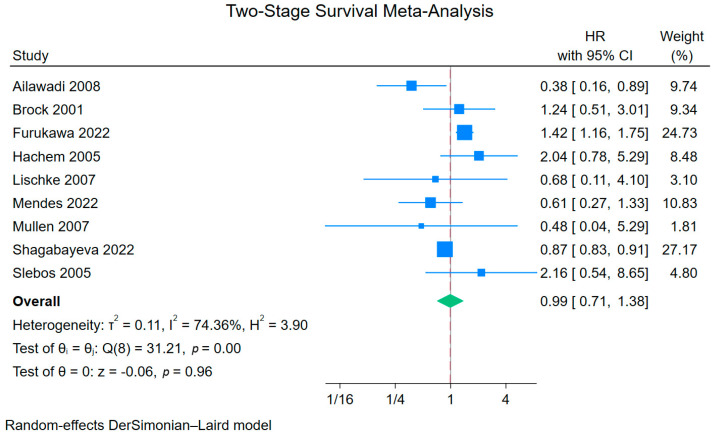
Two-stage meta-analysis for the primary endpoint (overall survival). Blue squares indicate study-specific hazard ratios with 95% CIs; the green diamond represents the pooled random-effects estimate and its 95% CI. The grey vertical line denotes no effect (HR = 1), and the red dashed line indicates the overall pooled effect.

Table 2 summarizes the main findings of this meta-analysis. Regarding overall survival, nine Kaplan–Meier curves were processed, digitalized, and reconstructed. Patients who received IL2-AR induction demonstrated improved overall survival compared with the SOC group (HR 0.88; 95%CI 0.85–0.93; *p* < 0.01; Figure 2). However, in the two-stage meta-analysis, based on study-specific HRs, this association was no longer statistically significant (HR 0.99, 95%CI 0.71–1.38; *p* = 0.96; I^2^ = 74.4%; Figure 3).

Leave-one-out analysis showed that the pooled effect size fluctuated in statistical significance depending on the study removed (Supplementary Figure S2). Importantly, among the two studies using registry-based data, only Shagabayeva et al. reported overall survival. When this study was excluded in the leave-one-out analysis, the pooled HR shifted toward the null and lost statistical significance, effectively mirroring a sensitivity analysis that excludes registry-derived data. The funnel plot did not reveal significant asymmetry (*p* = 0.758—Supplementary Figure S3).

### 3.5. Secondary Outcomes

**Figure 4:**
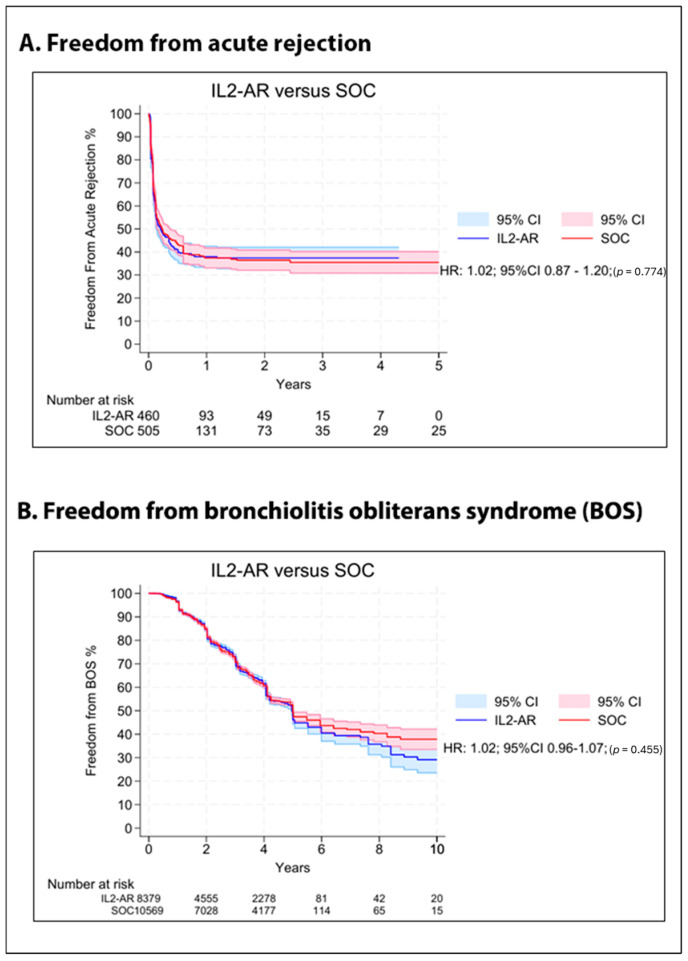
Overall Kaplan–Meier curves comparing IL2–AR versus SOC as induction therapy. (**A**) Freedom from acute rejection. (**B**) Freedom from bronchiolitis obliterans syndrome (BOS).

**Figure 5:**
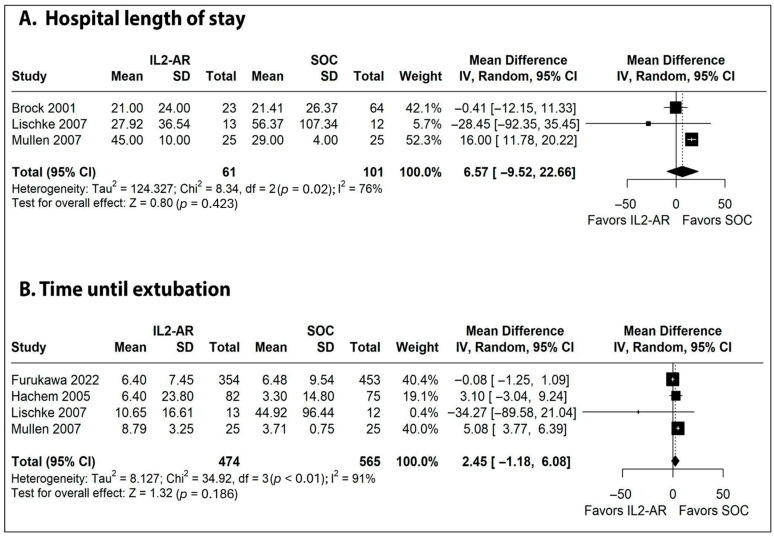
Forest plots of comparison between IL2–AR versus SOC as induction therapy in patients undergoing lung transplantation. (**A**) Hospital length of stay. (**B**) Time until extubation. Squares represent study-specific mean differences (size proportional to study weight) with horizontal lines indicating 95% CIs; the diamond represents the pooled random-effects estimate and its 95% CI. The vertical dashed line denotes the line of no effect (mean difference = 0). The plus sign marks the point estimate of individual studies when CIs are very narrow.

Related to freedom from acute rejection, the pooled analysis of seven Kaplan–Meier curves for the entire observation period showed no difference between the groups (HR 1.02; 95%CI 0.87–1.20; *p* = 0.774; Figure 4A). Regarding freedom from BOS, eight curves were pooled analyzed and revealed no difference between the groups (HR 1.02; 95%CI 0.96–1.07; *p* = 0.455; Figure 4B), as well as hospital LOS (MD 6.57 days; 95%CI −9.52 to 22.66; I^2^ = 76%; *p* = 0.423; Figure 5A) and time until extubation (MD 2.45 days; 95%CI −1.18 to 6.08; I^2^ = 91%; *p* = 0.186; Figure 5B).

## 4. Discussion

In this systematic review and meta-analysis of 12 studies including 27,855 patients, we comprehensively analyzed the outcomes of IL2-AR induction therapy compared to alternative induction agents or no induction therapy in lung transplantation. Our main findings were as follows: (I) IL2-AR induction was associated with higher overall survival compared to other protocols, but sensitivity analyses weakened the statistical significance; (II) no significant differences was observed in freedom from acute rejection; (III) no significant differences were noted in freedom from BOS, hospital LOS and time until extubation.

Lung transplantation frequently represents the final therapy option for patients with end-stage lung disease to enhance their quality of life and prolong survival [1,2]. Despite its benefits, the procedure remains associated with considerable morbidity and mortality, particularly due to complications related to acute and chronic lung allograft dysfunction [5]. Induction therapy, particularly with IL2-AR agents (Daclizumab and Basiliximab), has emerged as a promising strategy to optimize the immunologic response in lung transplant recipients, potentially improving graft survival while minimizing side effects [6,7]. Daclizumab was widely used in the past; however, in the last two decades, it was substituted by Basiliximab due to its simplified two-dose regimen, superior safety profile and comparable efficacy [7,28].

Our meta-analysis revealed that IL2-AR therapy was associated with improved overall survival compared to the control group, but sensitivity analyses weakened the statistical significance, warranting further exploration. The largest study included, by Shagabayeva et al. [6], demonstrated that induction therapy improved survival compared to no induction. However, when comparing different induction therapy protocols, results varied across studies. Furukawa et al. [22] and Hachem et al. [18] reported better survival rates in the Alemtuzumab and ATG groups, respectively, than the IL2-AR (Basiliximab) group, whereas Ailawadi et al. [19] showed the opposite. In a two-stage meta-analysis, the individual HR of the included studies did not retain significance and leave-one-out analysis revealed that the overall effect size alternated in statistical significance when individual studies were excluded.

Several factors may explain these inconsistencies. Most included studies were observational and limited by small sample sizes, leading to imprecision and potential confounding. Additionally, the largest dataset, by Shagabayeva et al. [6], relied on the United Network for Organ Sharing (UNOS) registry, which, although valuable, may not fully represent global practices or immunosuppression protocols. From a clinical perspective, determining the optimal induction strategy remains complex. Beyond induction, maintenance immunosuppression exerts a substantial influence on graft survival, acute rejection, and the development of bronchiolitis obliterans syndrome (BOS), and likely contributes to the variability observed across studies [29].

In a global analysis, it is important to consider the costs related to the induction therapy. Mullen et al. [13] demonstrated no significant differences in hospital-related costs between IL2-AR and ATG groups, even after excluding outliers with prolonged recoveries. In contrast, Shagabayeva et al. [6], in 2022, highlighted the cost-effectiveness of Basiliximab, estimating net savings of $3696.72 per patient and a cost-effectiveness ratio of $3723.23 per life year gained. These savings were attributed to reduced hospital stays, decreased acute rejection rates, and avoided procedures.

Regarding freedom from acute rejection, our results showed no significant differences between IL2-AR therapy and other induction protocols or no induction therapy. As observed with overall survival, the findings varied across the literature and depending on the induction protocol utilized. For example, Hachem et al. [24] reported better freedom from acute rejection rates in the ATG group compared to the IL2-AR group, whereas Ailawadi et al. [19] observed the opposite. Notably, freedom from acute rejection was not reported in the largest studies, such as Shagabayeva et al. [6] and Hachem et al. [24], resulting in a more evenly distributed weight among the included studies. This could reflect variability in maintenance immunosuppression and prophylaxis strategies across studies, which play critical roles in rejection prevention.

Furthermore, the absence of significant differences in secondary outcomes, including freedom from BOS, hospital LOS, and time until extubation may be influenced by patient-specific factors, surgical complexity, and center-specific protocols, potentially overshadowing the effects of induction therapy. In addition to freedom from BOS, this is shaped not only by induction therapy but also by long-term maintenance strategies.

Heterogeneity among studies is a key consideration, influenced by variability in patient demographics, clinical protocols, and adjunct therapies, which likely contributed to differences in reported outcomes. The lack of standardized definitions and inconsistent reporting of acute rejection and BOS further complicated the interpretation of our findings. Additionally, the ROBINS-I assessment indicated a moderate risk of bias, reinforcing the need for standardized definitions and protocols in future studies to reduce variability and enhance comparability.

Despite these limitations, our findings highlight the importance of re-evaluating IL2-AR therapy in lung transplantation. Clinical practice guidelines should focus on refining patient selection criteria and integrating IL2-AR therapy into comprehensive immunosuppressive regimens, aiming to strike a balance between prevention and infection risk. Future randomized controlled trials with standardized methodologies and more diverse populations are needed to confirm these findings and provide robust evidence for optimizing immunosuppressive protocols.

### 4.1. Implications for Clinical Practice and Future Research

The findings of this meta-analysis have relevant implications for clinical practice in lung transplantation. Although IL2-AR induction therapy was associated with improved overall survival in the primary one-stage analysis, the loss of statistical significance in sensitivity analyses warrants cautious interpretation. These results suggest that IL2-ARs may be an appropriate induction option in selected patients, but do not support their universal superiority over alternative induction strategies or no induction therapy.

These findings reinforce the importance of individualized decision-making when selecting induction strategies, taking into account recipient risk profile, institutional experience, and integration with maintenance immunosuppression rather than assuming a uniform benefit across all patients.

From a research perspective, our findings emphasize the need for prospective, multicenter studies with standardized definitions of rejection and BOS, as well as consistent reporting of induction strategies and long-term time-to-event outcomes. Fully stratified reporting of IL2-ARs, alternative induction agents, and no induction therapy would enable more robust comparative analyses. Future studies should also incorporate safety and cost-effectiveness outcomes to better inform clinical decision-making.

### 4.2. Study Strengths and Limitations

This study is the first meta-analysis to comprehensively evaluate IL2-AR induction therapy in lung transplantation. A key strength is the use of individual patient data from Kaplan–Meier survival curves, which enhanced the robustness of our long-term survival analysis. However, several limitations should be noted. The observational nature of most included studies introduces a risk of confounding, and variations in induction protocols and baseline characteristics contributed to heterogeneity. Additionally, it should be stressed that the inclusion of registry data reduces the reliability of the findings, and there was considerable variability in control regimes across the included studies.

Secondary outcome variability, limited generalizability, and the inability to fully assess adverse effects further highlight areas for future research. Rigorous, multicenter trials with standardized interventions are essential to establish the safety and efficacy of IL2-AR therapy in lung transplantation.

## 5. Conclusions

This meta-analysis shows that IL2-AR therapy may be associated with improved survival after lung transplantation; however, evidence remains inconclusive due to heterogeneity and limitations in study design. Furthermore, IL2-AR induction therapy did not demonstrate significant differences in freedom from acute rejection or secondary outcomes such as freedom from BOS, hospital LOS, and time until extubation.

## Figures and Tables

**Table 1:** Study and patient’s baseline characteristics of included studies.

Study	Time Frame	Type of Study	Country	IL2-AR	Control	Sample Size IL2-AR/SOC, *n*	Age IL2-AR/SOC, Median or Mean	Male IL2-AR/SOC, *n* (%)	Double Lung Transplant IL2-AR/SOC, *n* (%)
Ailawadi, 2008 [19]	1998–2006	Retrospective	USA	Daclizumab	ATG	98/65	52.7/53.9 *	55 (56%)/30 (46%)	26 (26.5%)/8 (12.3%)
Brock, 2001 [20] ✧	1995–1999	Prospective	USA	Daclizumab	ATG/OKT3	23/34/30	52/51/51 †	14 (61%)/16 (47%)/14 (47%)	7 (31%)/7 (21%)/13 (43%)
Burton, 2006 [21]	1992–2003	Retrospective	Denmark	Daclizumab	ATG	169/166	55/50 *	75 (44.4%)/72 (43.4%)	37 (21.9%)/68 (41%)
Furukawa, 2022 [22]	2011–2020	Retrospective	USA	Basiliximab	Alemtuzumab	351/452	60.16/60.23 *	210 (59.3%)/252 (55.6%)	306 (86.4%)/395 (87.2%)
Garrity, 2001 [8]	1996–1998	Retrospective	USA	Daclizumab	NI	27/34	48/49 †	10 (37%)/14 (41%)	14 (52%)/24 (71%)
Hachem, 2005 [23]	2000–2003	Retrospective	USA	Basiliximab	ATG	82/75	55.2/53.1 *	74 (90%)/71 (95%)	NR
Hachem, 2008 // [24]	2000–2004	Retrospective	USA	Basiliximab	ATG/NI	1124/597/2249	55/55/55 *	575 (51%)/297 (50%)/1155 (51%)	692 (62%)/323 (54%)/882 (39%)
Lischke, 2007 [25]	2002–2003	Prospective	Czech Republic	Daclizumab	ATG	13/12	46/40 †	8 (61.5%)/6 (50%)	6 (41.2%)/5 (41.7%)
Mendes, 2022 // [26]	2016–2019	Retrospective	Portugal	Basiliximab	ATG/NI	61/43/20	52/52/52.5 *	40 (65.6%)/27 (62.8%)/15 (75%)	50 (82%)/29 (67.4%)/16 (80%)
Mullen, 2007 [13]	2001–2003	RCT	Canada	Daclizumab	ATG	25/25	53/52 †	13 (52%)/15 (60%)	19 (76%)/18 (72%)
Shagabayeva, 2022 § [6]	2006–2018	Retrospective	USA	Basiliximab	Alemtuzumab/ATG/NI	11,045/1556/1421/8003	60/61/58/59 *	6584 (59.6%)/885 (56.9%)/813 (57.2%)/4844 (60.5%)	8008 (72.5%)/1140 (73.3%)/1021 (71.9%)/5110 (63.9%)
Slebos, 2005 [27]	1990–2001	Retrospective	Netherlands	Basiliximab	ATG	17/34	45.1/43.1 †	9 (52.9%)/18 (52.9%)	10 (58.8%)/28 (82.3%)

§ Data are presented for groups IL2-AR/Alemtuzumab/ATG/NI, respectively. // Data are presented for groups IL2-AR/ATG/NI, respectively. ✧ Data are presented for groups IL2-AR/ATG/OKT3, respectively. * Median. † Mean. ATG: Antithymocyte Globulin; SOC: standard of care; NI: no induction, NR: not reported.

**Table 2:** Summary of outcomes.

Outcome	Number of Studies	Number of Patients	Effect Estimate, Random Model (95% CI, *p*-Value)
Overall Survival	9	23,489	HR 0.88; 95% CI 0.85–0.93; *p* < 0.01
Acute rejection	7	878	HR 1.02; 95% CI 0.87–1.20; *p* = 0.774
Bronchiolitis obliterans syndrome	8	27,285	HR 1.02; 95% CI 0.96–1.07; *p* = 0.455
Hospital LOS	3	162	MD 6.57; 95% CI −9.52 to 22.66; *p* = 0.423
Time until extubation	4	1039	MD 2.45; 95% CI −1.18 to 6.08; *p* = 0.186

CI: confidence interval, HR: hazard ratio, MD: mean difference, LOS: length of stay.

## Data Availability

The data underlying this article are available in the article and in its online Supplementary Material.

## Supplementary Materials

The following supporting information can be downloaded at: https://www.mdpi.com/article/10.3390/jcm15041438/s1, Supplementary Figure S1: Critical appraisal of studies according to Risk Of Bias In Non-randomized Studies of Interventions (ROBINS-I); Supplementary Figure S2: Leave-one-out analysis for the primary endpoint (overall survival); Supplementary Figure S3: Funnel plot for the primary endpoint (overall survival); Supplementary Table S1: Complete search strategy.
