# Supplementary material for: Interleukin-2 Receptor Antagonist Induction Therapy in Lung Transplantation—A Meta-Analysis of Reconstructed Time-to-Event Data
Source: J Clin Med. 2026 Feb 12;15(4):1438. doi: 10.3390/jcm15041438 (PMC12942110; doi:10.3390/jcm15041438)

Supplementary Material

Supplementary Table S1. Complete search strategy.

Supplementary Figure S1. Critical appraisal of studies according to Risk Of Bias In Non-randomized Studies of Interventions (ROBINS-I)

Supplementary Figure S2. Leave-one-out analysis for the primary endpoint (overall survival).

Supplementary Figure S3. Funnel plot for the primary endpoint (overall survival)**.**

Supplementary Table S1. Complete search strategy

Search: ("lung transplant" OR "lung transplantation") AND ("induction therapy" OR "basiliximab" OR "daclizumab" OR "Interleukin-2 receptor" OR "Interleukin-2 inhibitors")<br><br>("lung transplant"[All Fields] OR "lung transplantation"[All Fields]) AND ("induction therapy"[All Fields] OR "basiliximab"[All Fields] OR "daclizumab"[All Fields] OR "Interleukin-2 receptor"[All Fields] OR "Interleukin-2 inhibitors"[All Fields])

Supplementary Figure S1. Critical appraisal of studies according to Risk Of Bias In Non-randomized Studies of Interventions (ROBINS-I)

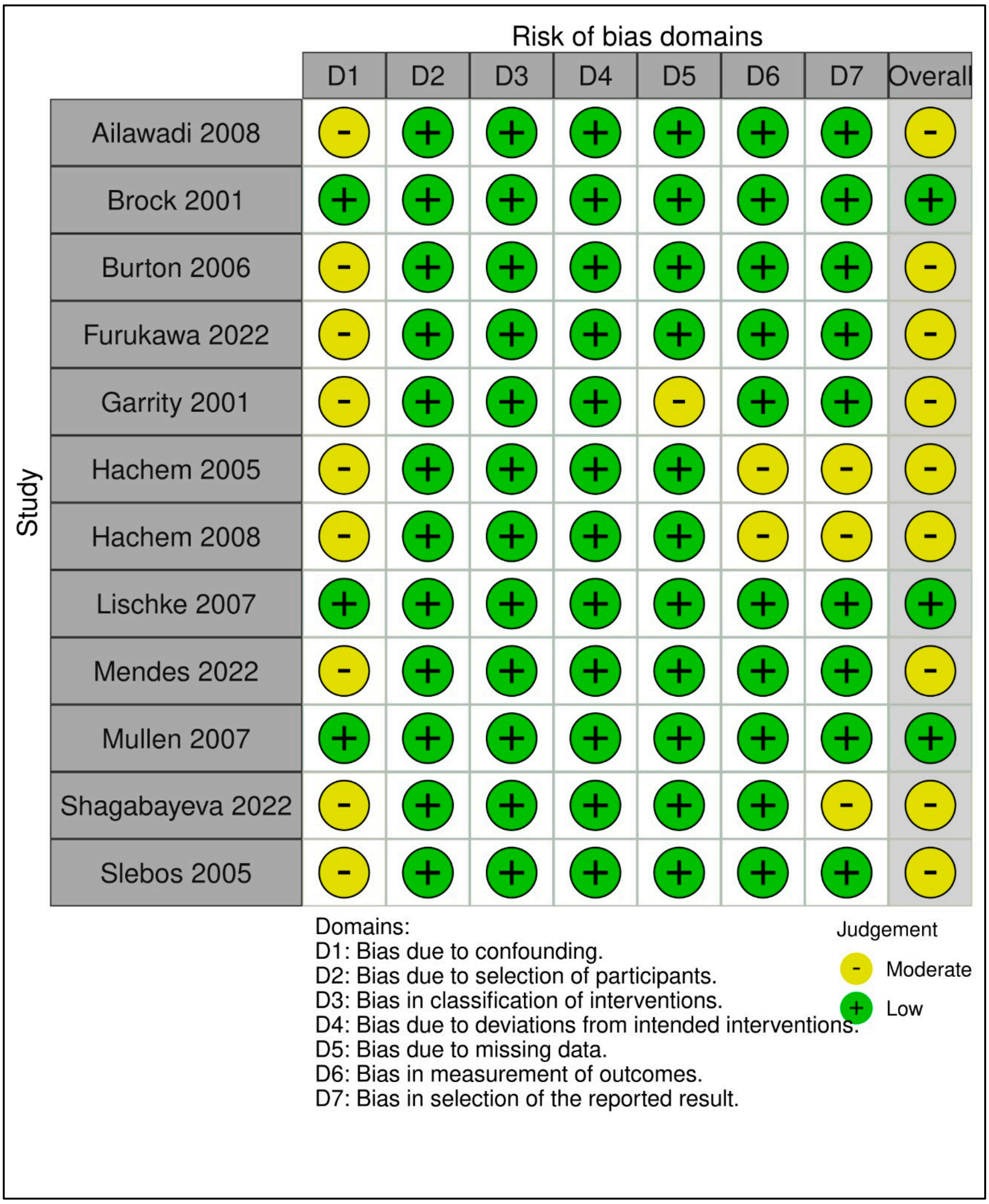

Supplementary Figure S2. Leave-one-out analysis for the primary endpoint (overall survival).

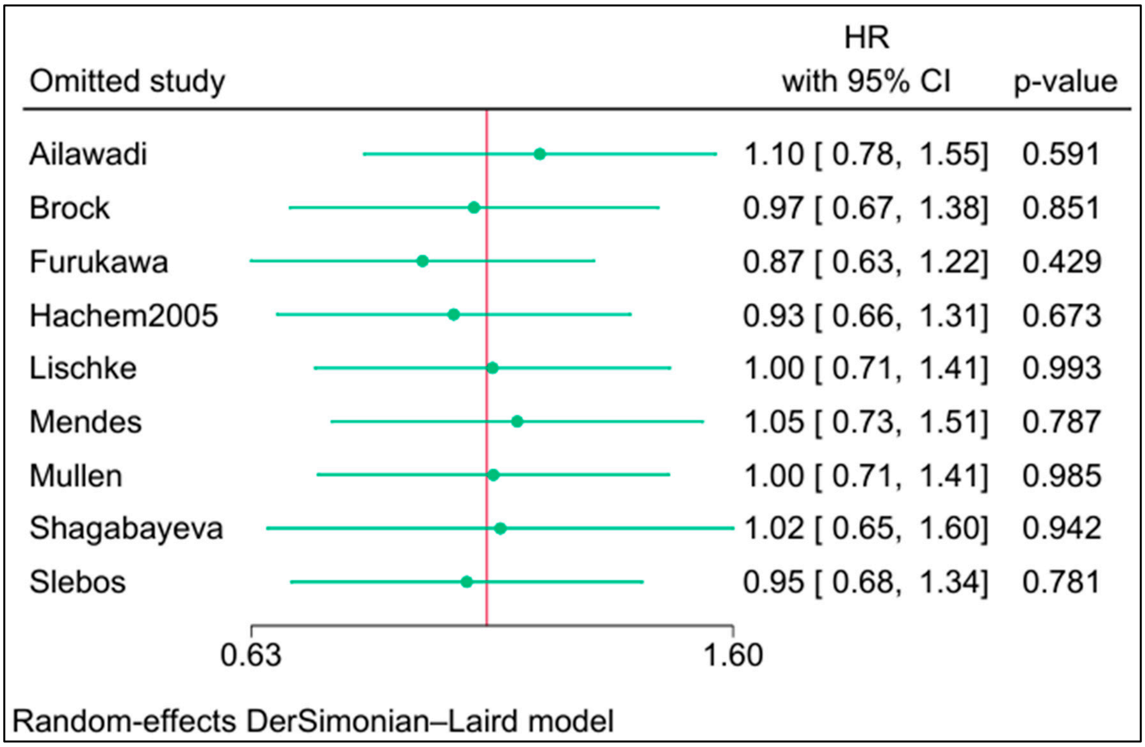

Supplementary Figure S3 - Funnel plot for the primary endpoint (overall survival).

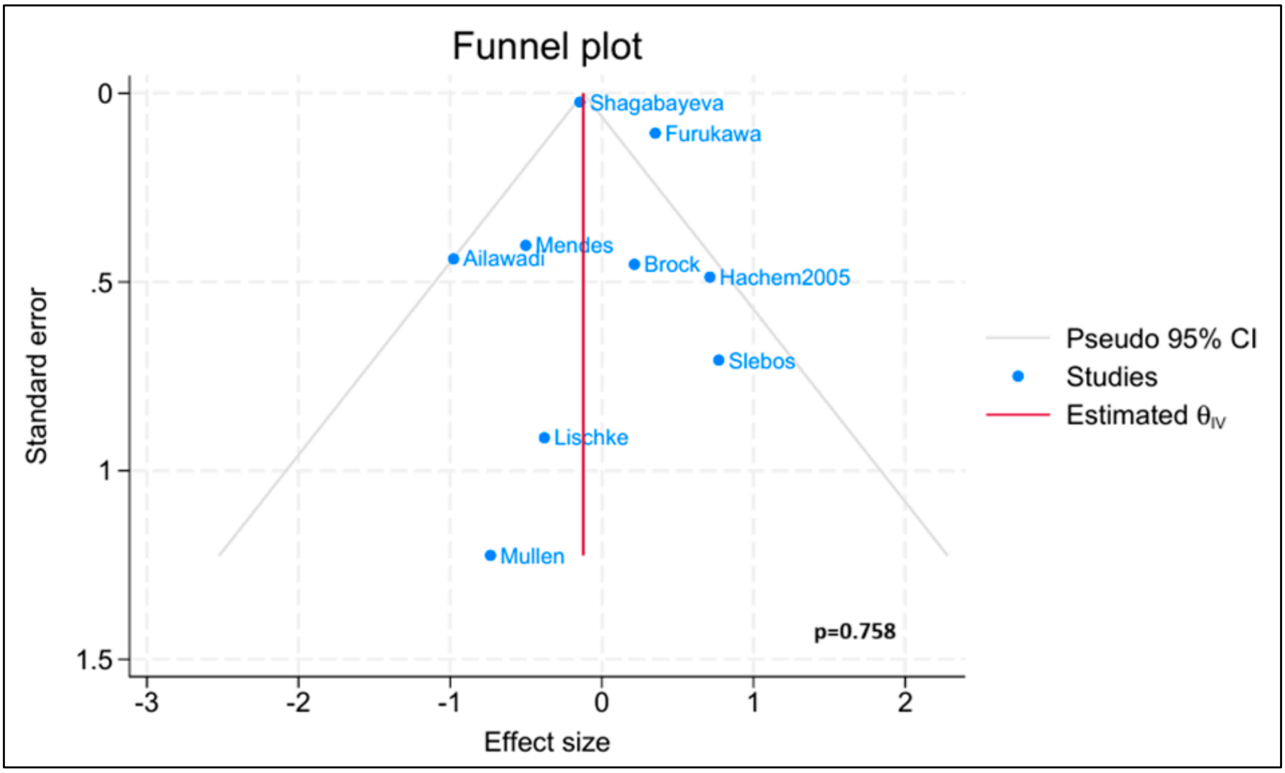

Supplement: Supplementary file 1 [file jcm-15-01438-s001.zip › jcm-4072068-supplementary.pdf]
